# *Haloferax volcanii* for biotechnology applications: challenges, current state and perspectives

**DOI:** 10.1007/s00253-019-10314-2

**Published:** 2019-12-20

**Authors:** R. U. Haque, F. Paradisi, T. Allers

**Affiliations:** 1 School of Life Sciences, Queens Medical Centre, University of Nottingham, Nottingham, NG7 2UH UK; 2 School of Chemistry, University Park, University of Nottingham, Nottingham, NG7 2RD UK; 3 Warwick Integrative Synthetic Biology Centre, School of Life Sciences, Gibbet Hill Campus, University of Warwick, Coventry, CV4 7AL UK; 4 Department of Chemistry and Biochemistry, University of Bern, Freiestrasse 3, 3012 Bern, Switzerland

**Keywords:** *Haloferax volcanii*, Biotechnology, Protein expression, Immobilization, Model organism

## Abstract

*Haloferax volcanii* is an obligate halophilic archaeon with its origin in the Dead Sea. Simple laboratory culture conditions and a wide range of genetic tools have made it a model organism for studying haloarchaeal cell biology. Halophilic enzymes of potential interest to biotechnology have opened up the application of this organism in biocatalysis, bioremediation, nanobiotechnology, bioplastics and the biofuel industry. Functionally active halophilic proteins can be easily expressed in a halophilic environment, and an extensive genetic toolkit with options for regulated protein overexpression has allowed the purification of biotechnologically important enzymes from different halophiles in *H. volcanii*. However, corrosion mediated damage caused to stainless-steel bioreactors by high salt concentrations and a tendency to form biofilms when cultured in high volume are some of the challenges of applying *H. volcanii* in biotechnology. The ability to employ expressed active proteins in immobilized cells within a porous biocompatible matrix offers new avenues for exploiting *H. volcanii* in biotechnology. This review critically evaluates the various application potentials, challenges and toolkits available for using this extreme halophilic organism in biotechnology.

## Introduction

Halophiles are extremophilic microbes that are widespread in all three domains of life—Eukarya, Bacteria and in particular, Archaea (Kamekura 1998; Oren 2013). They are found in hypersaline environments around the world where the salt concentrations is higher than sea water (around 3.5% dissolved salt), in various geographical niches such as salt lakes, deep sea, salt pans, saline soils, salt mines or salt marshes (Oren 2015). Based on their salt preference, halophiles can be split into three different categories: mild halophiles growing optimally at 1–3% (0.2–0.5 M) NaCl, moderate halophiles with optimum growth at 3–15% (0.5–2.5 M) and extreme halophiles with optimal growth at 15–20% (2.5–5.2 M) NaCl (Amoozegar et al. 2017; De Lourdes et al. 2013). Below 0.2 M NaCl (< 1%), microbes are classified as non-halophiles and a key difference is that the growth of mesophilic organisms is impaired at higher salinities, where archaeal species dominate. All halophilic archaea belong to the Euryarchaeota kingdom (Oren et al. 2009).

Halophiles have been important in natural salt production since they facilitate salt crystallization through the absorption of solar radiation using their carotenoid pigments, which confers distinct pink-reddish hues to the saltern crystallizer ponds they inhabit. More recently, halophiles have been used in biotechnology as producers of antimicrobials, bioplastics, biofuel, extremozymes, extracellular polysaccharides, retinal proteins, coloured pigments and compatible solutes (Amoozegar et al. 2019; Atanasova et al. 2013; Koller 2019; Patel and Saraf 2015; Torregrosa-Crespo et al. 2017). This has led to their applications in astrobiology, agriculture, food and nutrition industry, biosensor development and photochemical industry, bioremediation, textile and tanning industry, aquaculture, nanotechnology, sustainable chemical production and medical application (Le Borgne et al. 2008; Lee 2013; Margesin and Schinner 2001; Rodrigo-Baños et al. 2015). Several reviews on the biotechnologically valuable products generated by halophiles and their potential applications have been recently published (Antunes et al. 2017; DasSarma et al. 2010a; Mohammadipanah et al. 2015; Oren 2010; Yin et al. 2015).

Halophiles are under-represented in biotechnology compared with other extremophiles such as thermophiles or alkaliphiles, which have been used extensively for valuable product generation. This is surprising considering the biotechnological potential of halophiles and their great diversity across all three domains of life. While some halophiles are mechanically fragile, prone to lysis in low salt environment, and their culturing can lead to possible damage of the stainless equipment (due to the required molar concentrations of salt), some examples of their successful application in industrial biotechnology are represented by the application of purple membrane protein bacteriorhodopsin (from *Halobacterium salinarum*) in photochemical application, ectoine, an enzyme immobilizer used in the cosmetic industry (from *Halomonas elongata)* and β-carotene production (from green algae *Dunaliella salina)* (Margesin and Schinner 2001; Oren 2010).

*H. volcanii* is an extremely halophilic archaeon belonging to the family Haloferacaceae. Easy growth conditions, a short generation time and facile genetics have made it a model organism for haloarchaeal biology (Allers and Mevarech 2005; Leigh et al. 2011). It encodes numerous biotechnologically attractive halophilic enzymes with application potentials in sustainable fine chemical synthesis, bioprocessing, bioremediation, biofuel and bioplastic production (Amoozegar et al. 2019; Koller 2019; Lobasso et al. 2015; Timpson et al. 2013; Uthandi et al. 2009). Developments of tools such as bioreactor-scale overexpression of functionally active proteins and immobilization of cells within a porous matrix have opened up new possibilities in exploiting *H. volcanii* for biotechnological applications (Haque et al. 2019; Strillinger et al. 2016). This review summarizes the biotechnologically relevant features of this organism, the challenges involved and the tools available for realizing its true biotechnological potential.

## Genetics and biochemistry of *H. volcanii*

*H. volcanii* was first isolated from the sediment of the Dead Sea (Mullakhanbhai and Larsen 1975). The organism originally described as *Halobacterium volcanii* was named after the microbiologist Benjamin Elazari-Volcani who reported the presence of indigenous microbial life in the salt rich Dead Sea (Elazari-Volcani 1943). It grows optimally at 45 °C with a generation time of 2 h (Robinson et al. 2005). It is simple to culture in the laboratory since it grows aerobically in complex, minimal and synthetic media (Mevarech and Werczberger 1985). Compared with other extreme halophiles, *H. volcanii* tolerates a wide range of salt concentrations (1.8–3.5 M NaCl) (Allers 2010). It is comparatively resistant to contamination since few other organisms can survive the molar concentration of salt present in the growth media. It can grow up to a temperature of 50 °C (Dyall-Smith 2009). It lacks a rigid cell wall and instead presents a single layer of glycoprotein known as surface layer or S-layer (Rodrigues-Oliveira et al. 2017). Protein subunits in S-layers are held together by divalent cation such as Mg^2+^ (Cohen et al. 1991). Hence, the S-layer can be completely removed by treatment of *H. volcanii* with chelating agents such as EDTA.

The *H. volcanii* genome consists of a main chromosome (2.848 Mb) and three smaller mini-chromosomes (85.1 kb pHV1, 438 kb pHV3 and 636 kb pHV4) along with a 6.4 kb pHV2 plasmid (Hartman et al. 2010). It has a stable genome when compared with other extreme halophiles such as *Halobacterium salinarum*, which undergoes frequent IS-mediated rearrangement (López-García et al. 1995; Sapienza et al. 1982). One of the major incentives to exploit *H. volcanii* in biotechnology is it has an extensive genetic toolbox to perform a plethora of genetic manipulations. Transformation of DNA into *H. volcanii* is achieved using the PEG protocol (Cline et al. 1989). The method was further improved by generating strains deleted for the *mrr* restriction endonuclease gene, which reduces the transformation efficiency by cleaving the incoming foreign DNA at methylated sites (Allers et al. 2010). The *mrr* deficient *H. volcanii* strain allows direct and efficient transformation, eliminating the need to passage the foreign DNA in an *Escherichia coli dam* mutant. There are multiple shuttle vectors available for use, which are based on pHK2, pHV2 and pHV1 origins (Allers and Mevarech 2005; Allers et al. 2004). The ability of *H. volcanii* to grow in minimal and synthetic media has led to the development of multiple successful auxotrophic selection markers, where genes involved in amino acid or nucleotide biosynthetic pathways are used to complement chromosomal mutations. Uracil (*pyrE2*), leucine (*leuB*), thymidine (*hdrB*) and tryptophan (*trpA*) based selection markers have been used extensively for performing gene deletion/complementation in *H. volcanii* (Allers et al. 2004; Bitan-Banin et al. 2003; Leigh et al. 2011). A CRISPRi toolkit is also available for gene interference based on the clustered regularly interspaced short palindromic repeats (CRISPR)-Cas system (Maier et al. 2019). Gene expression reporters based on β-galactosidase and green fluorescent protein (GFP) are available (Holmes and Dyall-Smith 2000; Reuter and Maupin-Furlow 2005). For protein production, both inducible and constitutive promoter systems have been developed (Haque et al. 2019; Large et al. 2007), and metal based protein purification is commonly used owing to purification tags in the expression vectors (Allers et al. 2010). Given these genetic tools and a wide range of salt tolerance, it is not surprising that *H. volcanii* has been used as a heterologous host to study genes from other halophiles. For example, gas vesicles produced by the closely related species *Haloferax mediterranei* have been characterized using *H. volcanii* as a heterologous host, which is naturally devoid of gas vesicle genes (Pfeifer et al. 2002).

## Biotechnological potential of *H. volcanii*

Due to their remarkable properties such as antioxidants, colouring and light absorption, carotenoids are of great interest in biotechnology and medicine (Eggersdorfer and Wyss 2018; Fiedor and Burda 2014; Zhang et al. 2014a). Recently, there has been growing interest in naturally derived carotenoids owing to the increasing demand for bioactive compounds of natural origin with higher bioaccessibility and environmentally benign bioprocessing methods (Giani et al. 2019). The carotenoid biosynthesis pathway in *H. volcanii* begins with a condensation of two C_20_ molecules into C_40_ phytoene, a reaction catalysed by phytoene synthase, encoded by the *crtB* (HVO_2524) gene. Subsequently, phytoene is converted to lycopene by the phytoene desaturase enzyme encoded by *crtI* (HVO_2528). In *H. volcanii,* the *lye* (HVO_2527) gene encoded lycopene elongase subsequently converts lycopene to bacterioruberin. *H. volcanii* predominantly produces the C_50_ carotenoid bacterioruberin (82%) and its derivatives such as monoanhydrobacterioruberin (7%), bisanhydrobacterioruberin (2%) and dihydromonoanhydrobacterioruberin (2%), along with small amount of lycopene (0.3%) (Rønnekleiv 1995). A recent study has shown that bacterioruberin produced by *H. volcanii* is three times more effective as an antioxidant compared with β-carotene and improves the cell viability, motility and velocities of ram sperm cells (Zalazar et al. 2019). *Hv*LON3 strain, a conditional *H. volcanii* mutant for Lon protease produces 10–15 times more bacterioruberin than the parental strain given that Lon protease degrades the phytoene synthase, the rate limiting enzyme in carotenoid biosynthesis (Cerletti et al. 2018). *H. volcanii* is incapable of naturally producing β-carotene since the lycopene cyclase (encoded by *crtY*) is absent, whereas other halophiles such as *Haloarcula marismortui* and *Halobacterium salinarum* possess *crtY* (Baliga et al. 2004; Peck et al. 2002). However, the carotenoid biosynthetic pathway in *H. volcanii* can be genetically manipulated to produce β-carotene through the heterologous expression of *crtY* from *Haloarcula marismortui* (Smith and Ron 2018). Compared with other halophilic archaea, *H. volcanii* is better suited for heterologous pathway engineering due to its faster growth, readily available genetic toolkit and stable genome (Leigh et al. 2011). Given the absence of a rigid cell wall, *H. volcanii* affords a cheap and simple protocol for carotenoid extraction compared with the lengthy and complicated extraction procedure from pink shrimp or carrot, which involves the use of expensive equipment (Mezzomo et al. 2011; Rawson et al. 2011; Zalazar et al. 2019). Hence, by remodelling the carotenoid biosynthetic pathway through genetic engineering, production and extraction of β-carotene using *H. volcanii* may provide a more effective, efficient and affordable method of natural carotenoid synthesis than those currently offered by chemical synthesis route or by extraction route from other microbes, plants and algae.

Enzyme mediated biocatalysis offers a more cost-effective and environmentally sustainable way of generating biotechnologically valuable chemicals when compared with traditional chemical syntheses (Hughes and Lewis 2018). *H. volcanii* possesses alcohol dehydrogenase (ADH) enzymes, which catalyse the production of important building blocks in fine chemical, flavour and fragrance industries such as chiral alcohols (Chapuis and Jacoby 2001; Schmid et al. 2001). It encodes two haloalkaliphilic and thermoactive ADHs: *Hv*ADH1 (HVO_2428) and *Hv*ADH2 (HVO_B0071), which catalyse the reversible oxidation of primary and secondary alcohols into aldehydes and ketones, and the reduction of aldehydes and ketones into their corresponding alcohols in the presence of NADPH/NADH cofactors (Timpson et al. 2013). *Hv*ADH2 is industrially more attractive compared with *Hv*ADH1 due to greater enzyme activity, dual cofactor and broad substrate scope, stability in organic solvents and halophilic and thermophilic conditions (Alsafadi and Paradisi 2013; Timpson et al. 2013). Given its activity in high temperature (> 80 °C), it is described as the most thermoactive haloarchaeal ADH to date (Cao et al. 2008; Timpson et al. 2012; Timpson et al. 2013). It accepts a broad range of substrates ranging from medium chain alcohols to branched and aromatic alcohols for the oxidative reaction. For the reduction reaction, it accepts a range of aromatic ketone substrates such as alkyl, methyl and alpha-halo aryl ketones (Alsafadi et al. 2017). Immobilization within epoxy resin and metal organic framework material further improves the enzyme activity over a broader pH range and temperature and enhances enzyme stability in organic solvents (Alsafadi and Paradisi 2014; Carucci et al. 2018). It is also possible to improve the substrate scope of *Hv*ADH2 for biocatalysis through rational in silico enzyme design (Cassidy et al. 2017). *H. volcanii* expression systems can be used to overexpress and characterize industrially attractive enzymes from other halophiles (e.g. *Hm*ADH12 from extreme halophile *Haloarcula marismortui* and *Hs*ADH2 from the halophilic archaeon *Halobacterium salinarum* NRC-1) using the tryptophan inducible expression system (Liliensiek et al. 2013; Timpson et al. 2012). The same expression system has also been successfully used to produce haloarchaeal transaminase capable of producing chiral amines (Kelly et al. 2019). In this work, the authors overexpressed a haloarchaeal transaminase from a Triassic period halite deposit that showed halophilic activity, thermo and organic solvent tolerance profile.

Lignin and its derivatives are considered as serious contaminants in paper and pulp industries, tanneries and textile mills due to their poor biodegradability and intense colour (Costa et al. 2017; Raghukumar et al. 2008). Given that waste streams in these industries contain high concentrations of recycled salt, halophilic enzymes capable of lignin degradation could play a pivotal role in biopulping and biobleaching processes (Amoozegar et al. 2019). Laccase is a multicopper oxidase enzyme that couples oxidation of phenolic compounds to the four-electron reduction of molecular oxygen to water (Solomon et al. 1996). It has received attention in the biofuel industry due to its catalytic properties and helps to break down lignocellulose materials, prominent feedstocks for advanced biofuel production (Kudanga and Le Roes-Hill 2014). It has also been implicated in the detoxification of water and soil, in bilirubin level determination in serum and for use in biosensors and bioreactors (Sakurai and Kataoka 2007). *H. volcanii* encodes LccA (HVO_B0205), a rare archaeal laccase, which is secreted into the culture medium as a highly stable glycoprotein that is active at high salt concentrations (0.1 to 1.4 M) and temperature (55 °C). It accepts a broad range of organic substrates such as bilirubin, syringaldazine (SGZ), 2,2-azino-bis-(3-ethylben- zothiazoline-6-sulfonic acid) (ABTS) and dimethoxyphenol (DMP) and retains its activity in organic solvents such as dimethyl sulfoxide (DMSO) and dimethylformamide (DMF) (Uthandi et al. 2009). Hence, the LccA enzyme could serve as valuable biotechnological tool for the removal of phenolic contaminants in phenol-laden saline wastewater derived from various industrial settings (Moussavi et al. 2010). Despite the potential of LccA in biofuel industry, its direct in vivo lignolytic activity on lignin has still to be experimentally verified (Brink et al. 2019).

As can be seen from the above examples, enzymes from *H. volcanii* show polyextremophilicity, meaning that they are not only active in high salt environments but also are functional at increased temperature and in organic solvents. It has been established that enzymes from halophiles show greater activity and stability in organic solvents compared with their non-halophilic counterparts (Margesin and Schinner 2001). This unusual ability to be active in environments with low water activity (high salt, solvent and/or desiccation) has made *H*. *volcanii* and its enzymes ideal candidates for biotechnology advances.

Halocins are antimicrobial compounds naturally produced by halophilic archaea and bacteria living in hypersaline environments (Shand and Leyva 2007). These compounds are believed to confer selective advantages to the organism for survival in a competitive environment, including against other halophiles (Atanasova et al. 2013). Due to the emergence of antimicrobial resistant pathogens, there is a dire need to screen and characterize novel antimicrobials (Baker 2015). This has included the search for halocins that possess a broad range antimicrobial spectrum. *H. volcanii* KPS1 strain produces halocin that is active at high temperatures (up to 80 °C) and over a broad range of pH (3.0–9.0) and salinity (Kavitha et al. 2011). Most importantly, it shows antimicrobial activity against both Gram-positive and Gram-negative bacteria. Large amounts of salts are used in tanning processes, which provides a favourable environment for the growth of halophiles. Given its anti-halophilic activity, halocins can be exploited in the textile industry to inhibit the unwanted growth of halophiles that could be damaging for the product (Birbir et al. 2004). The ability of *H. volcanii* to degrade aromatic hydrocarbons such as anthracene, naphthalene, phenanthrene, pyrene and benzoanthracene could also be beneficial for bioremediation in hypersaline environments (Bonfa et al. 2011).

Polyhydroxyalkanoates (PHA) are polyesters composed of hydroxy fatty acids, which serve as intracellular storage material of carbon source and energy (Rehm 2007). They are found in a variety of microbes including haloarcheal species belonging to the genera *Haloarcula*, *Haloferax*, *Halococcus*, *Haloquadratum*, *Halobacterium*, *Haloterrigena* and *Natronobacterium* (Koller 2019). These water-insoluble nanosized cytoplasmic compounds crystallize after solvent extraction and most importantly, exhibit thermo-plastic and elastomeric properties (Poli et al. 2011). Therefore, PHA compounds have received global attention due to their importance as natural, bio-based, biocompatible and biodegradable alternatives to plastics of petrochemical origin. Poly 3-hydroxybutyrate (PHB) is the best known polyhydroxyalkanoate (PHA). Polymerization of 3-hydroxybutyryl-CoA results in PHA formation through PHA synthase activity (Rehm 2003). *H. volcanii* produces PHB up to 7% of its cell dry weight when grown on media containing 250 g/L salts, 10 g/L glucose and 1 g/L yeast extract (Fernandez-Castillo et al. 1986). An additional report for *H. volcanii* mediated PHA accumulation from sugarcane bagasse substrate, a fibrous leftover by-product of the sugarcane industry, was recently published (Salgaonkar and Bragança 2017). In contrast, another study reported no detection of PHB from *H. volcanii* (Legat et al. 2010). *H. volcanii* has been successfully used as a heterologous host to generate PHA through the expression of PHA synthase genes from the halophile *Haloarcula hispanica* (Han et al. 2009).

Alongside facile genetics, at least three other traits make *H. volcanii* an attractive host for bioprocessing. Firstly, industrially desirable quasi-sterile conditions are readily achieved, since few other microbes cannot grow in the molar concentrations of salt present in growth media for *H. volcanii*. Secondly, the cost involved in product extraction is significantly reduced since it is easy to lyse *H. volcanii* cells through addition of water and/or the use of simple chelating agents to disrupt the outer S-layer. Finally, the potential for LPS (lipopolysaccharide) mediated toxicity caused by bacterial hosts is eliminated by the use of *H. volcanii*. These properties would substantially facilitate downstream processing in economic and environmental terms during bioprocessing.

The S-layer of *H. volcanii* is a two-dimensional array of protein or glycoprotein subunits, which form part of the cell envelope components (Tamir and Eichler 2017). The ability of isolated archaeal S-layer proteins to assemble into regular arrays in liquid or on surfaces such as metals, polymers or interfaces such as lipid films and liposomes makes them attractive for potential applications in nanobiotechnology. These arrays show reproducible physiochemical properties down to the nanometre scale (Debabov 2004). The pores in S-layers are identical in size and morphology, and functional groups such as carboxylic acid, amine and hydroxyl groups on the protein lattices and pore areas are aligned in defined positions and orientations. These combined features have led to their applications in the production of isoporous ultrafiltration membranes, bioanalytical sensors, biomimetics, affinity membranes, immunotherapy, drug delivery, vaccine development and immobilization matrices for binding of functional molecules such as enzymes and antibodies (Ilk et al. 2008; Sleytr et al. 2007). Mammalian olfactory receptor from rat has been heterologously expressed in the lipid bilayer of the *H. volcanii* (Lobasso et al. 2015). Therefore, the lipid matrix of *H. volcanii* could serve as an ideal biomaterial for the future development of nanovesicle based hybrid biosensor devices.

## Challenges of applying *H. volcanii* in biotechnology

Halophiles adapt to high salt environment using two alternative strategies (Oren 2008). The first strategy, termed ‘salt-out’, is mainly adopted by halotolerant bacteria and eukaryotes. Excess salt is excluded from halotolerant bacteria and eukaryotes, with an increase in compatible organic solutes such as glycerol or glycine betaine in the cytoplasm to maintain an osmotic balance. These compatible solutes do not interfere with intracellular enzymatic activity. The second strategy, termed ‘salt-in’, is adopted mainly by haloarchaea including *H. volcanii.* Equimolar concentrations of salt, predominantly KCl, are accumulated in the cytoplasm to maintain the osmotic balance (Oren 2013). Since biosynthesis of organic osmotic solutes in the ‘salt-out’ strategy is energetically costly compared with the ‘salt-in’ strategy, it is haloarchaea such as *H. volcanii* that thrive in hypersaline conditions (Oren 1999). Halophilic proteins from *H. volcanii* have evolved to function in the presence of molar concentration of salt, they have an overall increase in the number of highly positively charged acidic residues on surface to make the proteins soluble in high salt environment, along with a decrease in overall hydrophobicity through the substitution of large hydrophobic residues on the surface with small hydrophilic ones (Danson and Hough 1997; Mevarech et al. 2000; Wright et al. 2002).

This peculiarity poses a challenge for the expression of active halophilic proteins in industrially attractive heterologous hosts such as *E. coli*, since these proteins often misfold and aggregate in low ionic strength environment. However, there have been a few reports of heterologous expression of halophilic proteins in *E. coli* (Cao et al. 2008; Cendrin et al. 1993; Domenech and Ferrer 2006; Johnsen and Schönheit 2004), which rely on solubilization of inclusion bodies followed by laborious solubilization and refolding of insoluble proteins in a hypersaline environment (Connaris et al. 1999; Singh and Panda 2005). An additional caveat is the requirement for prior knowledge of the target protein characteristics. In any case, this approach is not universally effective, as many enzymes remain inactive. For example, enzymatically active protein could not be recovered from the heterologously overexpressed alcohol dehydrogenase (*Hm*ADH12) of the extreme halophile *Haloarcula marismortui*, despite repeated attempts to solubilize the inclusion bodies in varying salt and pH conditions (Timpson et al. 2012). In another study, no protein expression was observed following attempts of overexpression in *E. coli* of two halophilic alcohol dehydrogenases (ADH/D1 and ADH/A1) from the deep Red Sea brine pools (Strillinger et al. 2016). These drawbacks of heterologous expression have led to the development of halophilic systems to generate biotechnologically important proteins that are functionally active.

One of the major obstacles in exploiting extremozymes is the low yield or activity of enzymes when cultivated on a large bioreactor scale, as required for industrial applications (Elleuche et al. 2014; Sarmiento et al. 2015). For example, it is essential to produce active alcohol dehydrogenase (*Hv*ADH), laccase (LccA) enzymes and bacterioruberin and halocins proteins (Table 1) from *H. volcanii* on large scale to realize their true biotechnological potential. It is challenging to ensure expression and purification of bulk quantities of highly active proteins in a halophilic environment. Co-purification of unwanted non-specific proteins with the target protein is often encountered by the researchers following affinity tagged protein purification (Kimple et al. 2013). A bottleneck of large-scale protein overexpression in *H. volcanii* host is their intrinsic ability to form biofilms (Chimileski et al. 2014; Frols et al. 2012), which can interfere with expensive sensors present in the bioreactor during the fermentation process and may also alter the characteristics of expressed proteins. Molar concentrations of salt required to culture *H. volcanii* can rapidly corrode stainless-steel bioreactors. This necessitates the use of reactors made of alternative materials rather than the readily available stainless-steel.

**Table 1:** Biotechnologically important components encoded by *H. volcanii*

Component	Potential applications	References
Phytoene synthase (HVO_2524), phytoene desaturase (HVO_2528) and lycopene elongase (HVO_2527)	Carotenoid biosynthesis	(Smith and Ron 2018; Zalazar et al. 2019)
Alcohol dehydrogenases-*Hv*ADH1 (HVO_2428) and *Hv*ADH2 (HVO_B0071)	Fine chemical production through biocatalysis	(Timpson et al. 2013; Alsafadi et al. 2017; Haque et al. 2019)
Laccase (HVO_B0205)	Biopulping, biobleaching, biofuel production	(Uthandi et al. 2009)
Halocin	Antimicrobials, bioremediation	(Kavitha et al. 2011)
Polyhydroxyalkanoate	Bioplastic production	(Koller 2019)
Outer surface layer (S-layer)	Nanobiotechnology	(Ilk et al. 2008; Sleytr et al. 2007; Lobasso et al. 2015)

## Tools for *H. volcanii* applications in biotechnology

**Fig. 1:**
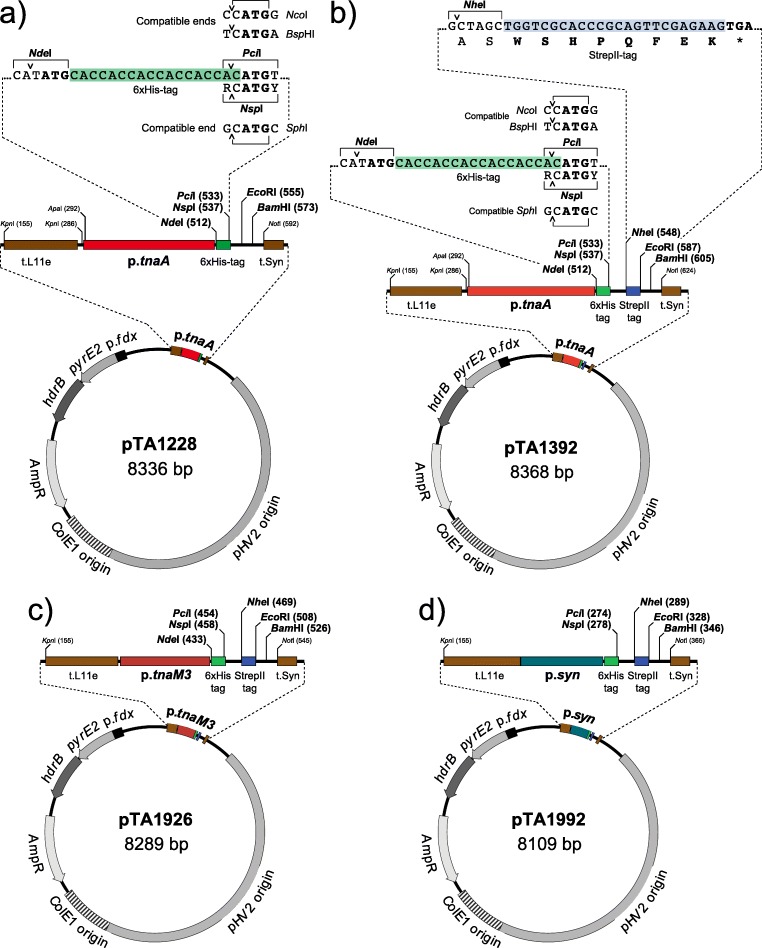
Regulated and constitutive protein expression system in *H. volcanii.***a** pTA1228. The p.*tnaA* promoter is induced by L-tryptophan and allows for regulated protein expression. An N-terminal histidine (His) tag is available for immobilized metal-based affinity chromatography (IMAC) protein purification. A multiple cloning site is located after His tag. At the 5′ end of the halophilic gene, the ATG start codon is replaced with an *Nco*I, *Bsp*HI, *Pci*I or *Sph*I site (depending on the coding sequence) and inserted in-frame with the His tag at the *Pci*I or *Sph*I site; at the 3′ end of the gene, an *Eco*RI or *Bam*HI site is used. The cloning cassette is flanked by L11e and t.Syn terminators to prevent read-through transcription. **b pTA1392.** A derivative of pTA1228. A C-terminal streptadivin II (StrepII) tag is available along with the His tag. At the 3′ end of the gene, the stop codon is replaced with an *Nhe*I site and inserted in-frame with the StrepII tag. **c pTA1926.** A 50% reduced strength tryptophan inducible promoter, when compared with p.*tnaA*. Both His and Strep II tags are available. **d) pTA1992.** A strong p.*syn* promoter for constitutive protein expression with both His and Strep II tags

For protein overexpression, a modified strain *H. volcanii* host strain H1424 (Δ*pyrE*2 Δ*hdr*B Δ*mrr Nph-pit*A *cdc48d-Ct*) is available along with its corresponding plasmid pTA1228 (Fig. 1a) (Brendel et al. 2014; Stroud et al. 2012). Regulated gene expression systems enable the tailoring of expression of biotechnologically important enzymes/proteins according to the industrial need. The pTA1228 plasmid (Fig. 1a) contains the tryptophan inducible promoter p.*tnaA* for protein expression (Large et al. 2007), which is active in the presence of >1 mM tryptophan. The plasmid pTA1228 also encodes an N-terminal hexahistidine (6xHis) tag to facilitate protein purification using immobilized metal-based affinity chromatography (IMAC), since this affinity tag is compatible with high salt media. The host strain H1424 is deleted for the histidine-rich PitA and Cdc48d proteins, major contaminants of the His-tagged proteins purified from *H. volcanii* using IMAC (Bab-Dinitz et al. 2006). Given the essential nature of the *pitA* and *cdc48d*, it was not possible to delete these genes completely; therefore, *pitA* was replaced with an orthologue (*Nph-pitA*) from the haloalkaliphile *Natronomonas pharaonis* that does not contain a high number of histidine residues, and a truncated version of *cdc48d* coding for a protein (Cdc48d-Ct) lacking the histidine rich C terminus was engineered (Allers et al. 2010; Stroud et al. 2012). The expression cassette in pTA1228 is flanked by two transcriptional terminators (t.L11e and t.Syn) to prevent read through transcription (Fig. 1a), and the pHV2 replication origin maintains the plasmid at a copy number of 6 per genome (Allers et al. 2004). Incorporation of *pyrE2* and *hdrB* marker genes in pTA1228 enables selection on media lacking uracil and thymidine, respectively; the latter is lacking in rich media (*Hv*-YPC), facilitating maximal cell growth and protein overexpression (Allers et al. 2004; Bitan-Banin et al. 2003).

To give researchers more options for protein purification, another plasmid pTA1392 (Fig. 1b) is available that encodes a C-terminal Streptavidin II tag (Strep II) for protein purification, alongside the established N-terminal His-tag (Braun et al. 2019); both tags are compatible with high salt concentrations. For protein expression at lower levels, for example if the protein is toxic to the host cells, the pTA1926 plasmid (Fig. 1c) contains the p.*tna*M3 promoter that shows 50% reduced activity compared with p.*tnaA* promoter present in pTA1228 and pTA1392 (Braun et al. 2019). For constitutive overexpression of proteins that are not harmful to the host, the pTA1992 plasmid (Fig. 1d) contains the highly active p.*syn* promoter (Haque et al. 2019; Large et al. 2007). This plasmid has the potential to make biotechnological processes cost effective since its usage alleviates the need for repeated tryptophan supplementation into *H. volcanii* cultures, which is an expensive amino acid.

A simple tryptophan-inducible protein overexpression system was reported that allows halophilic protein overexpression using a stirred tank bioreactor system (Strillinger et al. 2016). This study overexpressed two thermohalophilic alcohol dehydrogenases (ADH/D1 and ADH/A1), which showed about 28 fold more enzyme activity when compared with the uncontrolled shaker flask cultivations. The use of bioreactors has inherent advantages such as control of pH, temperature, supply of oxygens and nutrient media during the fermentation process. To prevent biofilm formation inside bioreactors, a *H. volcanii* strain (H1895) that is deleted for the two essential genes for biofilm formation: an integral membrane protein (HVO_1033) and ATPase subunit (HVO_1034) of the archaeal pilus biosynthesis machinery could be exploited in biotechnology given its inability to form biofilm during protein overexpression (Strillinger et al. 2016; Tripepi et al. 2013; Tripepi et al. 2012).

Alternative bioreactors other than corrosion prone stainless-steel are available for large scale halophilic protein production. A stirred and aerated bioreactor made of corrosion resistant materials such as polyetherether ketone (PEEK), borosilicate glass or silicon nitrite ceramics has been successfully used to generate poly-L-glutamic acid (PGA) or poly -β-hydroxy butyric acid (PHB) from the cultures of extreme halophilic archaeon *Natrialba* spp. (Hezayen et al. 2000). Another extreme halophile *H. mediterranei* has been cultured using a bioreactor with parts made of PEEK and borosilicate glass (Lorantfy et al. 2014). However, these materials are costly and more work is necessary in designing more energy efficient, corrosion-resistant and cost-effective alternative bioreactors for culturing *H. volcanii*.

**Fig. 2:**
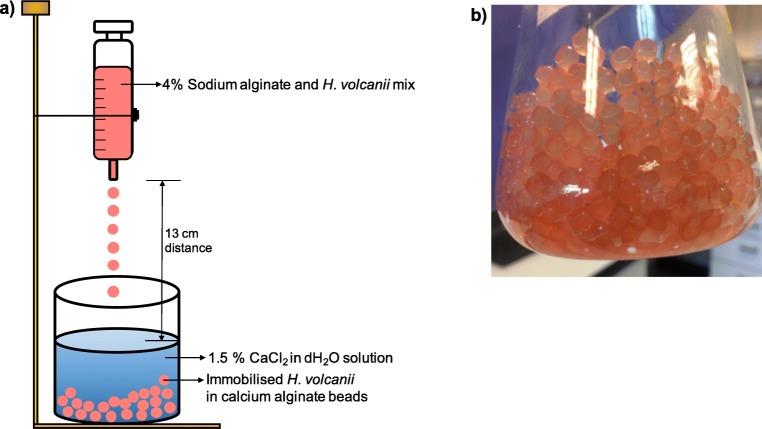
Simple method for immobilization of *H. volcanii* within the calcium alginate hydrogels. **a** Calcium alginate beads with encapsulated *H. volcanii* is formed instantaneously following the dropwise addition of resuspended *H. volcanii* cells in 4% sodium alginate solution into 1.5% CaCl_2_ solution. **b** Formation of pink beads due to the presence of high carotenoid pigment confirms entrapment of *H. volcanii* within the calcium alginate beads

Recent work has shown *H. volcanii* can be immobilized in inexpensive, non-toxic and highly porous calcium alginate beads following a simple method and used as whole cell biocatalyst (Fig. 2) (Haque et al. 2019). It offers halophilic enzyme expression with high product yield and reusability in a hypersaline environment. Furthermore, it provides data that calcium alginate beads with once immobilized *H. volcanii* can be used repeatedly for 12 successive biocatalytic cycles without compromising the bead morphology. Product yield from a batch of immobilized beads with *H. volcanii* that had been stored at room temperature for a month was very similar to freshly prepared beads. This system is particularly cost-effective with enzymatic processes that require expensive NADPH/NADH cofactor supplementation, since in situ cofactors generated by *H. volcanii* are used for the catalytic reaction, thus making the process self-sufficient. In this reported system, 1.1 g dry weight (from 120 ml broth culture) of immobilized *H. volcanii* whole cell biocatalyst showed high product yield range of 96.4–100% in the presence of 5 mM acetophenone substrate after 24 h. In contrast, a yield range of 48–94% was found from 500 ml of *Yarrowia lipolytica* in presence of 3.3 mM of the same substrate after 48 h (Janeczko et al. 2015). In another work, a product yield range of 53–63% was found with 5 g of the fungus, *Aspergillus niger* whole cell biocatalyst in the presence of 1 mM acetophenone substrate after 24 h (Kurbanoglu et al. 2007). Therefore, the performance of this immobilized *H. volcanii* whole cell biocatalyst is superior compared with the reported above two studies.

Immobilized *H. volcanii* within the calcium alginate beads offer multiple advantages for biotechnological applications (Fig. 2). Firstly, it stabilizes the whole cells within the calcium alginate bead matrix leading to higher cell density and specific productivity (Strand et al. 2004). Secondly, the porous alginate bead matrix eases the product separation and flow of material to the *H. volcanii* cells. Thirdly, this type of highly defined and reproducible encapsulation within the natural alginate biopolymer ensures biocompatible microenvironment for the maintenance of viable *H. volcanii* cells (Melvik and Dornish 2004). Finally, immobilized nature of the whole cells means it is ideal for their applications in bioreactors without the problem of cell wash out (Obradovic et al. 2004). Such simple system can easily be scaled up by packing the beads with immobilized *H. volcanii* inside a stirred tank fermenter (Strillinger et al. 2016). Organism such as *Alcaligenes faecalis* immobilized within the calcium alginate beads has been successfully used in packed bed bioreactor as whole cell biocatalyst (Xue et al. 2013; Zhang et al. 2014b). Intermittent or continuous substrate feeding approach using flow biocatalysis can be combined with in situ product removal strategy to further increase product yield and alleviate toxicity mediated by the presence of excessive substrate/product in the immobilized system (Freeman et al. 1993; Tamborini et al. 2018)). Therefore, if the biotechnological process involves supplementation or generation of a toxic compound, use of immobilized *H. volcanii* whole cell approach would be preferable over the bioreactor scale fermentation since it would offer more protection to the cells.

Interest in halophiles such as *H. volcanii* has led to the development of curated bioinformatic databases such as HaloWeb, HProtDB and HaloDom. These tools greatly accelerate bioprospecting through genome mining. HaloWeb (http://halo4.umbi.umd.edu) provides the complete genome sequences for all haloarchaeal genomes, along with BLAST options against various genomes and genomic maps (DasSarma et al. 2010b). HProtDB (http://webapp.cabgrid.res.in/protein/) contains information about physical and biochemical properties of halophilic proteins for 21 strains of Archaea and Bacteria (Sharma et al. 2014). HaloDom catalogues all the halophilic species studied to date across all three domains of life, Archaea, Bacteria and Eukarya (http://halodom.bio.auth.gr/). It also provides a ‘Halopredictor’ function to predict the signatures of halophilic adaptation in the FASTA sequence of a novel protein (Loukas et al. 2018). The ‘Halohandbook’ is a manual containing detailed laboratory protocols for studying halophiles including *H. volcanii* (Dyall-Smith 2009). It has been compiled with input from leading halophile researchers.

## Conclusions

Characteristics such as facile genetics, adaptation to low water environment and high salt tolerance have great potential in making *H. volcanii* a valuable resource for biotechnology. Of particular interest is the use of *H. volcanii* as a heterologous host to study important biotechnological enzymes from other halophiles, ranging from moderate to extreme. However, challenges still remain in applying *H. volcanii* in a broad range of biotechnological applications. With the discovery of novel biotechnologically important proteins, expansion of genetic toolkits and rapidly advanced technologies, the potential for exploiting *H. volcanii* in biotechnology can only grow.
